# Health research prioritization in Somalia: setting the agenda for context specific knowledge to advance universal health coverage

**DOI:** 10.3389/fpubh.2023.1202034

**Published:** 2023-11-09

**Authors:** Steven Ssendagire, Said Aden Mohamoud, Farah Bashir, Mohamed Amin Jamal, Mukhtar Bulale, Abdullah Azad, Marian Yusuf Warsame, Farhan Hassan, Mohamed Omar, Abdirizak Dalmar, Mary Joan Karanja, Lilly Muthoni Nyagah, Abdihamid Warsame, Abdifatah Ahmed Diriye, Sk Md Mamunur Rahman Malik

**Affiliations:** ^1^World Health Organization Country Office, Mogadishu, Somalia; ^2^Save the Children International, Mogadishu, Somalia; ^3^Somali Research and Development Institute, Mogadishu, Somalia; ^4^National Institute of Health, Mogadishu, Somalia; ^5^Department of Infectious Disease Epidemiology, London School of Hygiene and Tropical Medicine, London, United Kingdom

**Keywords:** Somalia, health, research, prioritization, universal health coverage

## Abstract

**Introduction:**

Despite recognition that health research is an imperative to progress toward universal health coverage, resources for health research are limited. Yet, especially in sub-Saharan Africa, more than 85% of the resources available for health research are spent on answering less relevant research questions. This misalignment is partially due to absence of locally determined health research priorities. In this study, we identified health research priorities which, if implemented, can inform local interventions required to accelerate progress toward universal health coverage in Somalia.

**Methods:**

We adapted the child health and nutrition research initiative method for research priority setting and applied it in 4 major phases: (1) establishment of an exercise management team, (2) a web-based survey among 84 respondents to identify health research questions; (3) categorization of identified health research questions; and (4) a workshop with 42 participants to score and rank the identified health research questions. Ethical approval was received from ethics review committee of the London School of Hygiene and Tropical Medicine (Ref:26524) and the Somali Research and Development Institute (Ref: EA0143).

**Results:**

Two hundred and thirty-one unique health research questions were identified and categorized under health systems, services and social determinants (77), communicable diseases (54), non-communicable diseases (41) and reproductive, maternal, new-born, child, adolescent health and nutrition (59). A priority score ranging from 1 to 9 was assigned to each of the questions. For each category, a list of 10 questions with the highest priority scores was developed. Across the four categories, an overall list of 10 questions with the highest priority scores was also developed. These related to bottlenecks to accessing essential health services, use of evidence in decision making, antimicrobial resistance, distribution and risk factors for non-communicable diseases, post-traumatic stress disorder and factors associated with low antenatal care attendance among others.

**Conclusion and recommendations:**

The developed priority research questions can be used to focus health research and to inform appropriation of health research resources to questions that contribute to generation of local health system knowledge which is required to accelerate progress toward universal health coverage in Somalia. The Somalia national institute of health should set up a consortium for provision of technical and financial support for research addressing the identified priority research questions, establish a mechanism to continuously monitor the extent to which new health interventions in Somalia are informed by knowledge generated through conducting prioritized health research and prioritize interventions aimed at strengthening the broader national health research system for Somalia.

## Introduction

The road to universal health coverage (UHC) is unique for every country and therefore requires interventions that are informed by context specific knowledge. The 2013 World Health Report clearly identifies health research as an imperative for accelerating progress toward UHC (1). The 3 key messages in this report are; (1) UHC cannot be achieved without evidence from research, (2) All nations should be producers of research as well as consumers, the creativity and skills of researchers should be used to strengthen public health programs, and (3) To make the best use of limited resources, systems are needed to develop national research agendas, to raise funds, to strengthen research capacity, and to make appropriate and effective use of research findings (1). Unfortunately, resources for health research are limited (2). Worse still, as reported in the 2014 by the Lancet series on how to increase value and reduce wastage in medical research, up to 85% of research conducted is wasted and do not contribute to systems strengthening, one of the major reasons explaining this wastage being that less priority health research questions are being addressed (3). This wastage is anticipated to be even greater in sub-Saharan Africa where the national health research systems are more fragile due to lack of appreciation for the importance of research for health, limited interaction between academia and health programmers, limited dissemination of research findings beyond journals, poor administrative support for research, bottlenecks related to inadequacy of manpower for health research, inaccessibility to new technology, tools and facilities for research, lack of functional research ethics review systems and limited investment of local resources in health research among others (4, 5). Approaches to strengthening health research have been framed under the broad context of a health research system defined as “the people, institutions and activities whose primary purpose is to generate high quality knowledge useful for the promotion, restoration and maintenance of the health status of populations including a mechanism to encourage the utilization of research” (6). A health research system is conceptualized as having four major building blocks (stewardship, financing, capacity building, and production and use of research) and nine operational components (6). Therefore, strengthening health research systems requires that bottlenecks in each of the nine operational components are identified and comprehensively addressed in a collaborative manner. The same framework has been operationalized and used to assess, monitor and/or evaluate health research system capacity development in Africa (7). One of the most critical operational components under the stewardship building block is to pinpoint the specific priorities for health research and remain faithful to the plan (2, 7).

Progressively, there has been notable improvement in health research system capacity development in Africa. However, a number of bottlenecks still exist across all nine operational components of health research systems in Africa (8, 9). Specifically, a survey on national health research systems strengthening conducted in 2018 by Rusakaniko and colleagues indicated that about 50% of the surveyed World Health Organization (WHO) African Region Office (AFRO) member states did not have national health research priorities (10). This is despite its critical importance in advancing health research in terms of effective resource allocation, aligning available research funds to burden of disease and addressing disparities in health research funding where up to 90% of global research funds are used to research health problems that effect less than 10% of the global population (4, 11).

Somalia is gradually emerging from a long period of conflict. Recovery of Somalia’s health system, including the national health research system, is nascent and can be traced back to the early 2010s. In a short time, from 2010 to date, a number of steps toward strengthening the country’s health research system, such as forging health research collaborations, with groups like the Somali-Swedish Researchers Association, and the establishment of the Somali Health Action Journal, have been implemented (12–16). A systematic assessment of national health research system capacities is key to comprehensive identification of bottlenecks that need to be addressed to improve the generation and use of research relevant to advancing UHC. Such an assessment is yet to be conducted for Somalia.

It is however promising that some research activity on Somali health issues is being conducted. For example, a review of publications on Somali health topics and local issues conducted on a sample of 304 publications from 1945 to 2022 revealed that: (1) 56–80% of the publications addressed communicable diseases; (2) 56–84% of the publications were authored by non-Somalis, (3) 81% of the first authors did not have any affiliation to an organization from Somalia; (4) 11% of the publications were funded by national sources; (5) 12% of data analysis for these publications was done in Somalia; and (6) 31–51% of the publications were original articles (16). To some extent, these findings speak of health research system ecosystem in Somalia. Broadly however, the extent to which the current health research in Somalia is relevant for Somalia was not known primarily because the priorities for health research, a key component of national health research systems, had not yet been developed for Somalia.

In this study, and within the broader framework of strengthening the Somalia national health research system, we described the methodology and the outcomes of a national health research prioritization exercise for Somalia.

## Methodology

### Study design

There are multiple methods that can be used for research priority setting. The common methods for research priority setting include; (1) Essential National Health Research (ENHR) approach, (2) Combined Approach Matrix (CAM), (3) James Lind Alliance Priority-setting Partnerships (PSPs), (4) Delphi techniques and Child Health and Nutrition Research Initiative (CHNRI). For our case, we opted for the CHNRI method. This is because compared to the other methods, the CHNRI method is regarded to be more integrative, more systematic, more flexible and more transparent (17, 18). In our case, the CHNRI method was adapted and implemented through four major phases: (1) establishment of the exercise management team, (2) identification of the health research questions; (3) categorization of identified health research questions and finally; (4) scoring and ranking of the identified health research questions.

### Implementation of the CHNRI method

#### Phase 1: establishment of the exercise management team

The conceptualization and implementation of the prioritization exercise was led by an exercise team composed of technical experts from the Somalia National Institute of Health (NIH), WHO Somalia country office, London School of Hygiene and Tropical Medicine, the Somali Research and Development Institute, and Somali National University and East Africa University. The team was established and led by NIH. Through weekly planning meetings, the management team designed and implemented a web-based survey to identify the possible health research questions, categorized the identified health research questions, developed criteria for scoring and ranking the identified health research questions, and finally organized and managed a 2-day workshop where participants applied the developed criteria to score and rank the identified health research questions.

#### Phase 2: identification of health research questions

The health research questions for Somalia were identified though a web-based survey. A web-based questionnaire was developed by the exercise management team using *Kobo Toolbox*; a free open-source tool for mobile data collection. The key question in the questionnaire was; identify three most important health issues that needed to be investigated and to transform the identified health issues into a research question. The face-validity of the questions was pre-tested among the exercise management team and adjusted accordingly. The questionnaire was not piloted because this was not deemed necessary. The web-based questionnaire can be accessed through the following link: https://ee.humanitarianresponse.info/x/TUa5CRbC. The targeted web-survey respondents were experts in health research in Somalia. These were identified through multiple methods. These included: (1) rapid review of online databases for research conducted in Somalia; (2) review of relevant government documents/reports; (3) review of the list of stakeholders who participated in the first research conference organized by the Somalia NIH; (4) purposive selection of renowned health researchers in Somalia; and (5) snowball sampling where renowned health researchers were asked to identify their fellows in Somalia. Through these strategies, a total of 200 health research experts in Somalia were identified. These were all invited to participate in the web-based survey through email. Purposive selection of experts is preferred over random selection of respondents for priority research setting exercises (19). The web-based questionnaire was sent to all the potential respondents by email. The questionnaire also collected the sociodemographic and research related characteristics of the respondents. Respondents were also required to indicate if they were interested in participating in a subsequent physical workshop to score and rank the identified research questions. Those interested in participating in the workshop also completed and send back a signed consent form. Respondents were required to respond to the questionnaire within 2 weeks after receiving the questionnaire or otherwise be considered as non-respondents. No specific number of responses (out of the 200) was targeted. All responses received within 14 days of sending out the invitation to potential respondents were analyzed. Web-survey responses received beyond 14 days of sending out the invitation to participate in the survey were excluded from the analysis. A total of 84 out of the 200 potential respondents completed the web-based questionnaire, representing a response rate of 42%. This meets the recommended optimum number of 30–50 experts required to result into a replicable prioritization exercise using the CHNRI method (20). The characteristics of the 84 respondents are detailed in Table 1. These identified a total of 263 health research questions.

**Table 1:** Characteristics of the 84 web-survey respondents and the 42 workshop participants.

Variable	Value	Web-survey (84)		Prioritization workshop (42)	
		Count	%	Count	%
Sex	Male	63	75%	27	64%
	Female	20	24%	15	36%
	Not disclosed	1	1%	0	0%
Level of education	Undergraduate degree	9	11%	2	5%
	Master’s degree	51	61%	33	78%
	PhD	18	21%	7	17%
	Postdoctoral	4	5%	0	0%
	Unspecified	2	2%	0	0%
Profession	Health worker	19	23%	30	71%
	Manager/coordinator	8	10%	5	12%
	Policy maker	2	2%	N/A	N/A
	Researcher	50	60%	N/A	N/A
	Others	5	5%	7	17%
Current employer	Federal and state MOH	8	9%	11	26%
	International NGO	8	9%	5	12%
	Professional body	4	5%	N/A	N/A
	University	47	56%	23	55%
	Research/policy institution	3	4%	N/A	N/A
	United Nations	8	9%	2	5%
	Others	6	7%	1	2%
Previous involvement in health research in Somalia	Yes	77	92%	N/A	N/A
	No	7	8%	N/A	N/A
Provided their identity (names and email)	Yes	29	35%	N/A	N/A
	No	55	65%	N/A	N/A
Interested in participating in the prioritization workshop	Yes	55	65%	N/A	N/A
	No	1	2%	N/A	N/A
	Did not indicate	28	33%	N/A	N/A
Publication experience	5 or more manuscripts	N/A	N/A	15	36%
	Less than 5 manuscripts	N/A	N/A	27	64%
Public health research experience	10 and more years	N/A	N/A	5	12%
	5–9 years	N/A	N/A	19	45%
	Less than 5 years	N/A	N/A	18	43%
Duration in current position	5 or more years	N/A	N/A	28	76%
	2–4 years	N/A	N/A	11	26%
	Less than 1 year	N/A	N/A	3	7%

#### Phase 3: categorization of identified health research questions

**Figure 1:**
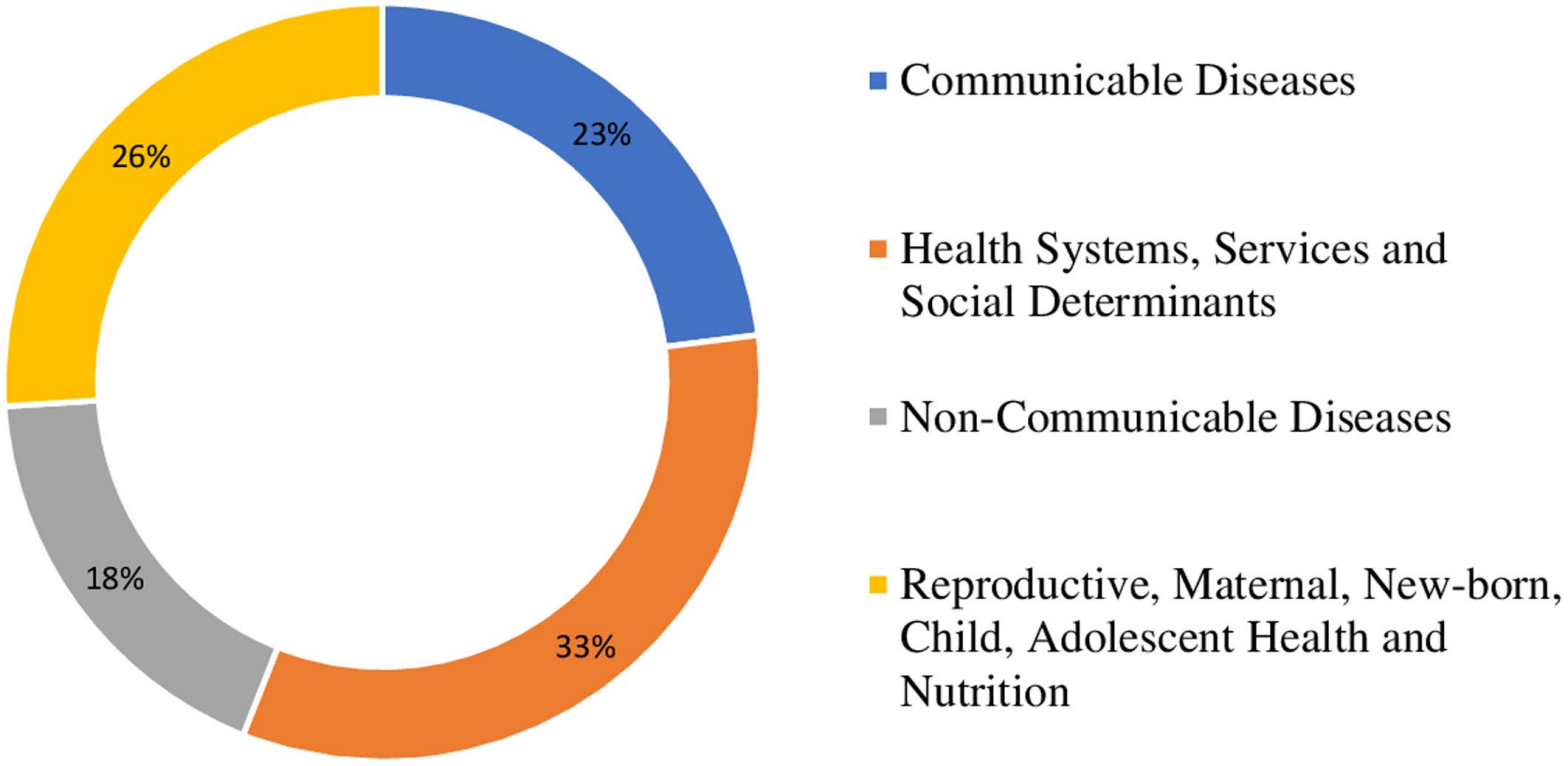
Pie chart showing the proportion of identified health research questions per category.

**Figure 2:**
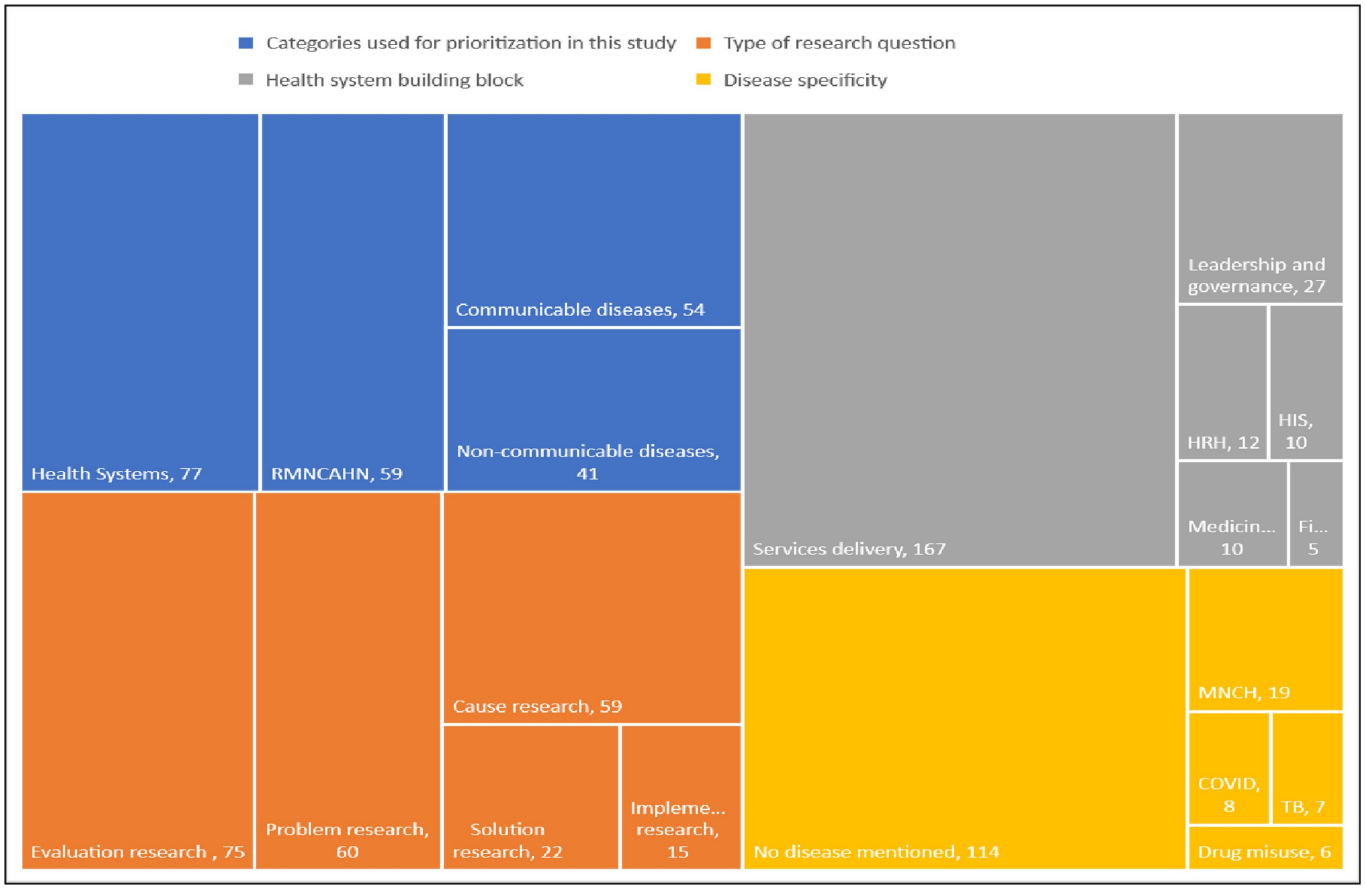
Treemap visualizing categorizations of identified health research questions.

Identified health research questions were reviewed by the prioritization exercise management team. Questions that were very similar to each other were merged. Duplicates were removed. A unique set of health research questions was retained. These were grouped under four thematic areas: (1) health systems, services and social determinants; (2) communicable diseases (CDs); (3) non-communicable diseases (NCDs); and (4) reproductive, maternal, new-born, child, adolescent health and nutrition. This is reflected in the pie chart in Figure 1. Other possible categorizations of the retained research questions are shown in the treemap in Figure 2.

#### Phase 4: scoring and raking of the identified health research questions

**Figure 3:**
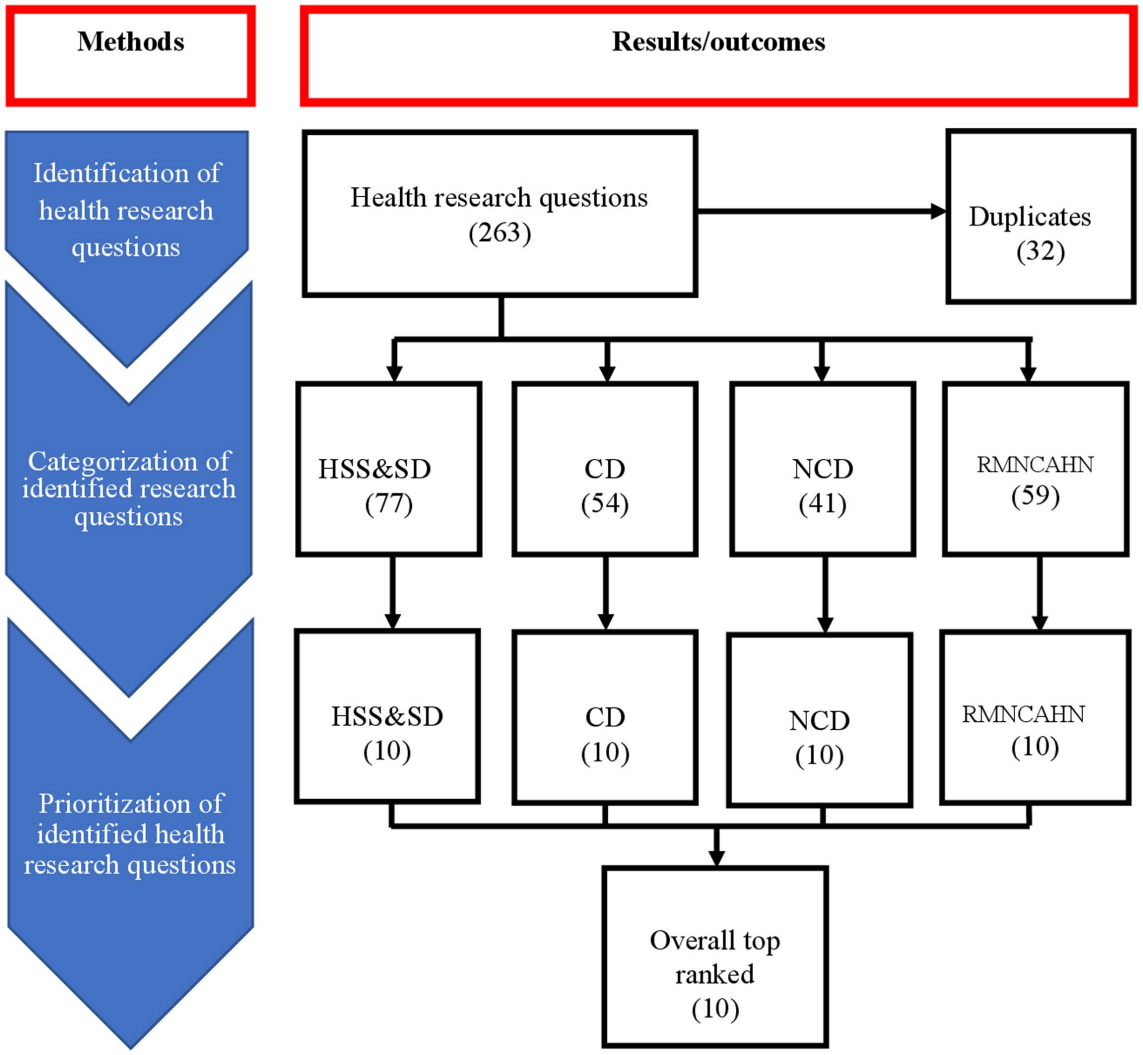
Flowchart of the health research prioritization process.

The scoring and ranking of the identified health research questions was done in a workshop setting. The workshop was held over 2 days. The workshop was organized and facilitated by the prioritization exercise management team described above. A total of 42 participants from organizations representing federal and state health authorities, local and international academic and research institutions took part in the workshop. The characteristics of the workshop participants are summarized in Table 1. The number of participants is consistent with the recommended number of experts to be consulted during priority setting exercises that apply the CHNRI method (20). Before the workshop, each of the prospective participants was assigned to one of the four groups corresponding to the four categories under which the 231 research questions had been grouped: (1) health system, services and social determinants of health; (2) CDs; (3) NCDs; and (4) reproductive, maternal, new-born, child, adolescent health and nutrition. During the workshop, each group was assigned two facilitators who had previously been guided on how to facilitate the scoring and ranking process. Each group was assigned to score and rank their respective research questions. Within a given group, participants individually scored each question on two criteria: (1) importance of the research question and (2) feasibility of the research question. The scoring scale for each criterion was 1–9, 1 corresponding to the lowest and 9 corresponding to the highest possible score for each criterion. For each participant, the final score for each question was the mean of the two scores for the two criteria. After each participant had individually scored each research question, using Excel software (Microsoft Corporation, Redmond, WA), the individual scores were pooled together, and a group mean score for each research question calculated. Each of the four groups sorted their research questions from the question with the highest group mean score to the question with the lowest mean score. Every group made a plenary presentation on their 10 research questions with the highest priority score, making a total of 40 research questions. Finally, using Excel, the 40 research questions were pooled together and sorted from the question with the highest to the question with the lowest group priority score. The top 10 research questions with the highest priority scores were selected to constitute the 10 priority health research questions for Somalia. This process is summarized in the flowchart in Figure 3.

### Ethical considerations

The health research prioritization exercise was considered as part of the broad program of approved work for organizations that led on the planning and implementation of the exercise. This withstanding, the following were undertaken to uphold the basic principles of research conduct: (1) the exercise received ethical approval from ethics review committee of the London School of Hygiene and Tropical Medicine (Ref: 26524) as well as the Somali Research and Development Institute (Ref: EA0143). Workshop participants chose to take part in the exercise voluntarily. Informed consent was obtained through returning a completed and signed consent form which was shared during the web-survey where possible research questions were identified. The methods and results presented here are devoid of individual participant identifiers. The final report of the prioritization exercise was shared among all key stakeholders involved in the generation and use of health research in Somalia. Multiple platforms will be used to disseminate the results of the prioritization exercise with institutions that conduct and support the conduct of health research in Somalia.

## Results

### Characteristics of the survey respondents and workshop participants

Eighty-four respondents completed the web-based survey for identifying health research questions for Somalia, representing a response rate of 42%. The majority of the survey respondents were male (75%), had a master’s degree (61%), employed with a university (56%) and were interested in participating in the prioritization workshop (65%). Of all the respondents, 42 took part in the 2-day workshop to score and rank the identified health research questions. Most of the prioritization workshop participants were male (64%), had a master’s degree (78%) and were health workers by profession (71%). Detailed characteristics of the web-survey respondents and workshop participants are provided in Table 1.

### Categorization of the 231 identified unique health research priorities

The 231 unique health research questions were categorized under, communicable diseases (CDs), health systems, services and social determinants (HSS&SD), non-communicable diseases (NCDs), and reproductive, maternal, new-born, child, adolescent health and nutrition (RMNCAHN). Most of the health research questions (77, 33%) were grouped under the category of HSS&SD (see Figure 1). The 231 unique questions were grouped under four categories: (1) HSS&SD (77), (2) CDs (54), NCDs (41), and RMNCAHN (59). The questions in each of these categories were scored on importance and feasibility. The 10 questions with the highest score were selected as the priority research questions for every category. These 40 were pooled and sorted. The 10 questions with the highest scores were selected as the overall leading health research priorities for Somalia. This process is summarized in a flow chat (see Figure 3).

### The 10 health research questions for each of the four thematic areas

For each thematic area, using group mean scores, the 10 highest ranking health research questions were selected. Under the category of HSS&SD, prioritized research questions focused on assessing the challenges and bottlenecks of delivering the essential package of health services to remote and hard-to-reach areas in Somalia and examining the current level on which research informs decision making and strategies employed to improve the uptake of research findings for the decision-making process. Under CDs, the top research questions focus on studying the magnitude and distribution of antimicrobial resistance of pathogenic bacteria and prevalence, severity and associated risk factors for dental caries among school children. Under NCDs, the top research questions relate to the identification, distribution and magnitude of NCDs among the Somali population and their associated risk factors, and, knowledge, attitude and perception, prevalence, and factors influencing obesity. Under RMNCAHN, prioritized research questions relate to factors contributing to preference of pregnant women on the home delivery over the institutional health delivery, and understand the factors associated with low completion rate of antenatal care attendance of pregnant women. The highest-ranking health research questions and their scores under each of the four thematic areas are summarized in Table 2.

**Table 2:** The priority health research questions for each of the four thematic areas.

Thematic area	Top 10 priority health research questions	Mean prioritization score
Health systems, services and social determinants	Late presentation for facility-based healthcare	9
	Access to the essential package of healthcare services among remote or rural communities	9
	Lessons learned from female health workers in Somalia	9
	Current mechanisms for interaction between researchers and decision makers in the generation of research evidence	9
	Impact of regular staff training on the quality of patient services	9
	Prevalence, severity and risk factors for dental caries among school children in Mogadishu, Somalia	8,5
	Patient privacy and confidentiality in private hospitals	8.5
	Major challenges for using data on decision making	8.5
	DHIS2 vs. other sources of data for informing decision making among doctors	8
	Prescription quality and associated practices among medical doctors	8
Communicable diseases	Role/use of culturally adapted and context specific approaches for COVID-19 prevention	8.5
	Pattern and extent of antimicrobial resistance of pathogenic bacteria in Somalia	8.5
	Prevalence of hepatitis B among pregnant and delivering women at selected health facilities in Somalia	8.5
	Burden of neglected tropical diseases	7.5
	Prevalence, severity and factors associated with dental caries among school children in Somalia	8.5
	Burden and factors associated with leading infectious diseases in Somalia	7.5
	Prevalence and factors associated factors with *Helicobacter pylori* infections in Somalia	7.5
	Hotspots for emerging/re-emerging infectious diseases of animal origin including Rift Valley fever, bovine tuberculosis and MERS-COV in Somalia	7
	Prevalence and factors associated with HIV infection in Somalia	6.5
	Coordination and capacity building to support cross-border disease surveillance and control policy and strategy	6.5
Non-communicable diseases	Magnitude of chronic kidney disease and its risk factors among patients attending selected public hospitals in Mogadishu, Somalia	8
	Prevalence and risk factors for NCDs	8.5
	Status of cancer management	8.5
	Prevalence and risk factors for hepatocellular, esophageal and breast cancers	8.5
	Prevalence and factors associated with overweight and obesity	8.5
	Availability and quality of facility-based mental health services	7.5
	Prevalence of post-traumatic stress disorder among displaced populations	7.5
	Prevalence and factors associated with drugs and substance abuse among school children	8
	Extent and impact of drug abuse among students	8
	Barriers and facilitators to implement high intensity talk therapy in community care	7
Reproductive, maternal, neonatal, child, adolescent health and nutrition	Preference of home delivery over health facility delivery	8.5
	Enrollment and retention into antenatal care clinics	8.5
	Effectiveness of campaigns against female genital mutilation practices	8
	Barriers to primary health care utilization	8
	Prevalence and associated factors associated with malnutrition among under five children	8
	Prevalence and factors associated with anemia among pregnant women	7.5
	Determinants and utilization of maternal and child health services	7
	Determinants (including male involvement) and utilization of contraceptives	7
	Immunization status and its determinant among 12–23 months children	7
	Utilization of sexual and reproductive health services	6

### The 10 overall prioritized health research questions

The 10 highest ranking health research questions for each of the four thematic areas were pooled together to make a total of 40 research questions. These were sorted according to their priority scores. From these, the 10 with the highest priority scores were selected as the overall 10 leading priority health research questions for Somalia. These are: (1) bottlenecks to accessing essential health services among hard-to-reach communities; (2) the status of using evidence-based research for decision making; (3) pattern and extent of antimicrobial resistance; (4) prevalence, severity and risk factors for dental caries among school children; (5) prevalence, distribution and risk factors for NCDs; (6) prevalence, risk factors, knowledge, attitude and perception toward adult obesity; (7) prevalence and factors associated with drugs and substance abuse among school children; (8) prevalence of post-traumatic stress disorder among displaced populations; (9) factors influencing preference of home delivery over health facility delivery; and (10) factors associated with low antenatal care attendance and completion.

## Discussion

### The importance of health research to UHC

Health research is vital in informing the development of technology, systems and services required to optimize utilization of quality health services by those who need them while at the same time reducing their risk of financial impoverishment. The technology, systems and services relevant in one context may however not be relevant in another context. In other words, the road to UHC is local. This calls for the creation of context specific information to guide decisions on which health services to deliver, to whom they should be delivered and how they should be delivered. These multiple health information needs can result into a multiplicity of health research questions. Because resources for health research are limited, there is a grave need to identify and prioritize health research that answers questions that are important and relevant to advancement of local priority health needs (21), which was done in this study.

### Alignment of identified research questions to Somalia’s UHC roadmap

Somalia has the lowest UHC index in the world standing at 22/100, indicating that only 22% of the country population has access to essential health services (22, 23). The index is calculated from indicators for measuring access to/satisfaction with services under the four thematic areas of; reproductive, maternal, neonatal, child and adolescent health (RMNCH), communicable diseases (CDs), non-communicable diseases (NCDs), and services, access and capacity (22, 23). These are the same areas with the highest disease burden in Somalia (24). In our study, we identified priority research questions and categorized them under similar thematic areas. The identified research questions directly relate to the thematic areas and indicators for monitoring UHC progress in Somalia and are therefore of utmost relevance to advancing the UHC agenda in Somalia (22, 23, 25). Conducting research to generate knowledge/evidence related to the identified priority questions will therefore directly improve UHC and the UHC score for Somalia.

### Methodological strengths and limitations

There are two major limitations that can potentially affect the validity of results from research prioritization exercises; transparency and appropriateness of those who score the identified/listed research questions.

#### Transparency

This relates to the clarity of how the identified/listed research questions are scored. Majority of methods and publications on research prioritization are not very explicit on how they scored the listed research questions. Our study was guided by the CHNRI method for research priority setting which among all methods for priority setting is the most elaborate on how to score identified/listed research questions (18, 26). In our methodology, we clearly state the 2 criteria (importance of the research question and feasibility of the research question) and the scale (1–9) which was used to score each question and how the final score for each question was generated (mean of the 2 scores for each criterion). According to WHO, the criteria against which identified research questions can be scored relate to public health benefit [potential return from conducting research on a given question or feasibility (whether the research is scientifically possible) or cost (availability of time, money, equipment and staff to implement the research question)] (27). As a study strength, both our criteria (importance and feasibility) perfectly align with the WHO guidance on how to choose criteria for research prioritization. However, these 2 criteria were pre-determined by the exercise management team without consulting potential exercise participants/respondents on which (any how many) criteria should be used. Stakeholder consultation could have resulted into a different set of scoring criteria which could potentially result into different scores which could also potentially result into a different list of health research priorities than the one developed in this study.

#### Appropriateness of the study respondents/participants

The other major potential limitation of research prioritization relates to whether respondents and/or participants in the prioritization exercise are appropriate/the right respondents/participants. The initial list of 231 health research questions was identified by a total of 84 survey respondents which is 42% of the 200 potential respondents to whom the survey questionnaire was sent. There is no published guidance on the optimum number of people from whom possible research questions should be solicited. What is perhaps more important is to ensure that the people who identify these research questions are indeed producers and users of health research and that all possible research questions have been identified (28). Our combination of both purposive sampling and snowball sampling is consistent with how sampling has been done in research prioritization exercises that employ the CHNRI method. Through this sampling procedure, we aimed to optimize the likelihood that surveyed respondents are health research producers or users (19, 29). For the prioritization workshop, we engaged with a total of 42 participants. This number is consistent with the recommended number of participants to engage in prioritization exercises that employ the CHNRI methodology (30). Finally, for the web-survey component, we achieved a response rate of 42%, which is above the average response rate (40%) for online surveys (31). To achieve this, we employed some of the known strategies for improving response rates during online surveys which include sending personalized invitations to potential respondents to complete the questionnaire, keeping the questionnaire simple, providing a long duration (2 weeks) within which respondents had to complete the questionnaire and sending constant reminders to potential respondents who had not completed the questionnaire (31, 32). For the web-survey, more than 85% of the respondents have a master’s degree and above, with 92% having previous research experience in Somalia. For the prioritization workshop, 57% of the participants had five or more years of experience with 76% having spent five or more years in their current positions and 90% having a master’s degree and above. Respondents/participants were also recruited from multiple sectors and multiple professions. A survey to characterize producers and users of health research in Somalia has not yet been conducted. However, the characteristics out study respondents/participants have been described in sufficient detail and our findings should be interpreted and generalized to the extent that the characteristics of our study respondents and the general producers and users of health research in Somalia are comparable.

A total of 231 unique health research questions were scored by 42 workshop participants. At the time of scoring, the 231 questions were put in 4 categories. Each category had between 77 and 41 questions. Also, the 42 workshop participants did not score each of the 231 questions, but instead, each participant scored only those questions in the category to which they were assigned. This minimized the likelihood of fatigue and misconduct during the scoring. There is no guidance on the appropriate number of questions that should be scored by each participant to reduce the likelihood of fatigue and misconduct. During the scoring, each of the 4 groups was closely supported by a team of 2–4 workshop facilitators which can minimize the likelihood of misconduct. Also, for each of the categories, we pooled all the individual scores and used these to generate a group score for every research question. This also can potentially even-out any misconduct if done by a few participants.

The decision to report on the top 10 health research priorities for each of the four thematic areas and the 10 overall research priorities is meant to focus attention to a few critical health research questions than looking everywhere. Elsewhere, similar exercises also often report the top 10 priority research questions (33, 34). Nonetheless, the fact that priority scores are available for each of the 231 research questions that were identified. It is possible to increase or even reduce the number of prioritized research questions.

## Conclusion

To the best of our knowledge, this was the first ever health research prioritization exercise for Somalia. Through the exercise, using the local knowledge of health system actors, 40 priority health research questions, 10 under each of the four thematic areas of (1) health systems strengthening and social determinants of health (HSS&SD), (2) communicable diseases (CDs), (3) non-communicable diseases (NCDs), and (4) reproductive, maternal, neonatal, child and adolescent health (RMNCH), were identified. The developed list of priority research questions can be used to focus research conducted institutions of higher learning and other research institutions in Somalia. The list can also be used to inform appropriation of financial resources for health research in Somalia thus avoiding the dedication of scarce resources to research whose results have little or no use in addressing the most urgent health knowledge/information needs of Somalia. These together with other health research system strengthening interventions will contribute to generation of local health system knowledge which is required to accelerate progress toward universal health coverage (UHC) in Somalia.

### Recommendations on future directions for health research in Somalia

We suggest that the Somalia NIH should work with the academia, research institutions and other relevant organizations in the country to:

1. Set up a consortium for provision of technical and financial support for research addressing the identified priority research questions.
2. Establish a mechanism to continuously monitor the extent to which ongoing research is addressing the identified priority research questions and to monitor the extent to which new health interventions in Somalia are informed by knowledge generated through conducting prioritized health research.
3. To prioritize interventions aimed at strengthening the broader national health research system for Somalia including strengthening legislation on health research, establishment of a national ethical review board, strengthening health research in local universities, supporting publication of local health research in peer reviewed journals and establishment of a health research knowledge translation platform.

## Data availability statement

The raw data supporting the conclusions of this article will be made available by the authors, without undue reservation.

## Ethics statement

The studies involving humans were approved by the ethics review committee of the London School of Hygiene and Tropical Medicine (Ref:26524) and the Somali Research and Development Institute (Ref: EA0143). The studies were conducted in accordance with the local legislation and institutional requirements. The participants provided their written informed consent to participate in this study.

## Author contributions

SS, SM, FB, MJ, MB, AA, MW, FH, MO, AD, LN, and AW conceived and designed the study. SM, SS, FB, MJ, MB, AA, MW, FH, MO, AD, MK, LN, AW, and AAD contributed to data acquisition and data analysis. SS drafted the manuscript. SM, FB, MJ, MB, AA, MW, FH, MO, AD, MK, LN, AW, AAD, and SM reviewed the manuscript. SS and AA revised the manuscript. All authors read, and approved the submitted version of the manuscript. All authors contributed to the article and approved the submitted version.
